# Simultaneous Multicompartmental Emphysema Following Orbital Wall Fracture: A Case Report and Literature Review

**DOI:** 10.7759/cureus.95088

**Published:** 2025-10-21

**Authors:** Abdul Rehman, Atta ur Rahman, Raja Ansar Hameed, Muhammad Ahmad Javed, Fazeelah Bibi, Syeda Zoya Chishti

**Affiliations:** 1 Emergency Department, St. Luke's General Hospital, Kilkenny, Kilkenny, IRL; 2 Emergency Department, Pakistan Institute of Medical Sciences Hospital, Islamabad, PAK; 3 Obstetrics and Gynaecology Department, Pakistan Institute of Medical Sciences Hospital, Islamabad, PAK

**Keywords:** blunt facial trauma, conservative management, medial orbital wall fracture, orbital compartment syndrome, orbital emphysema, orbital wall fracture, subconjunctival emphysema, subcutaneous emphysema

## Abstract

Orbital emphysema is a relatively common finding after orbital wall fractures, but the simultaneous presence of orbital, subcutaneous, and subconjunctival emphysema is rare and signals the presence of an underlying orbital wall fracture with possible sinus communication. We present the case of a 21-year-old male who sustained blunt trauma to the left side of his face during an assault, leading to periorbital bruising, subconjunctival emphysema, and a medial orbital wall fracture. Computed tomography confirmed emphysema involving the orbit, subconjunctival space, and subcutaneous tissues, with associated nasal bone fractures. Conservative management with oral antibiotics, topical ocular medications, and avoidance of nose-blowing led to full recovery without sequelae. This case highlights the importance of recognizing subconjunctival emphysema as a potential marker of orbital fractures and supports the role of conservative therapy in uncomplicated presentations.

## Introduction

Orbital emphysema is a well-recognized complication of orbital wall fractures, occurring in 20-60% of cases [1]. It results from the entry of air into orbital tissues through defects in the sinus walls [1]. While subcutaneous emphysema of the face and eyelids frequently accompanies orbital emphysema, subconjunctival emphysema is relatively rare and often overlooked due to concurrent periorbital soft-tissue swelling [2,3].

Most cases are self-limiting and resolve spontaneously as the entrapped air is gradually absorbed [4]. However, severe orbital emphysema may lead to orbital compartment syndrome with the risk of permanent vision loss from optic nerve ischemia; therefore, prompt recognition and exclusion of this possibility with appropriate investigations is crucial [4]. Reports of simultaneous orbital, subcutaneous, and subconjunctival emphysema remain scarce in the literature, with only two cases reported to date, to the best of our knowledge [5,6]. In this report, we describe a young male with a medial orbital wall and nasal bone fractures who developed combined orbital, subcutaneous, and subconjunctival emphysema. He was treated conservatively with complete recovery, contributing an additional case to the limited published evidence on this rare clinical scenario.

## Case presentation

A 21-year-old male presented to the emergency department (ED) with swelling and bruising around the left eye after being punched on the left side of his face. He reported a brief episode of epistaxis from both nostrils, which had resolved before arrival at the ED.

**Figure 1:**
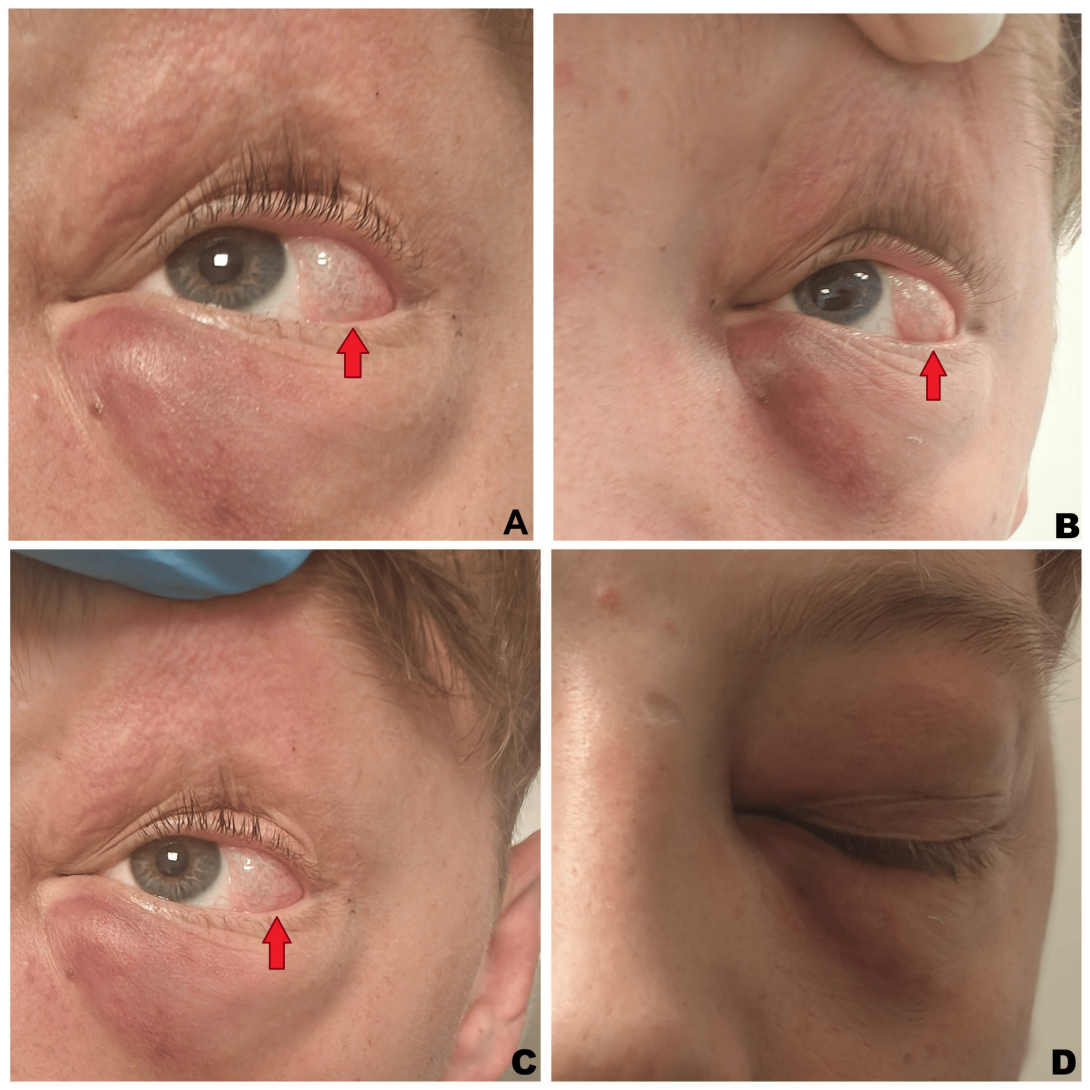
Clinical photograph of the patient at presentation showing left periorbital swelling, ecchymosis, and temporal subconjunctival emphysema (red arrows) with localized conjunctival elevation

On examination, there was left periorbital ecchymosis and a temporal subconjunctival cystic lesion with palpable crepitus, consistent with subconjunctival emphysema (Figure 1). Tenderness was noted over the left medial and inferior orbital walls, and crepitus was present in the left periorbital region as well as over both zygomatic areas. The nose was intact, with no evidence of septal hematoma or external deviation. No proptosis was observed.

Ophthalmic evaluation revealed equal and reactive pupils without a relative afferent pupillary defect. There was mild restriction of abduction in the left eye, but no diplopia or blurred vision was reported. Visual fields were full, and color vision (Ishihara testing) was intact. Intraocular pressure measured 12 mmHg in the right eye and 15 mmHg in the left. Both slit-lamp and fundus examinations were normal.

**Figure 2:**
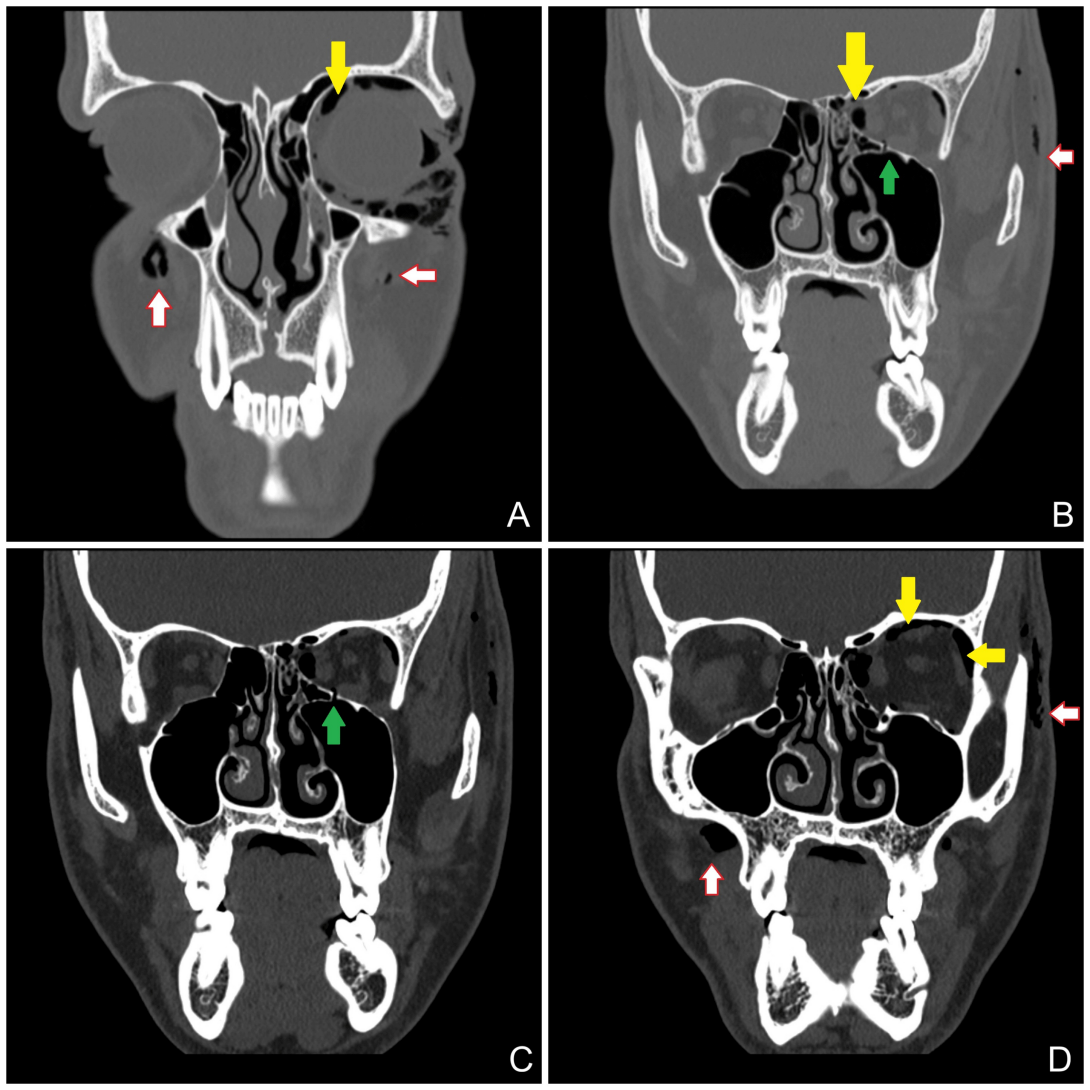
Coronal view computed tomography (CT) scan of the face demonstrating orbital (yellow arrows), subconjunctival, and subcutaneous emphysema (white arrows) associated with a small fracture (green arrows) of the left medial orbital wall

**Figure 3:**
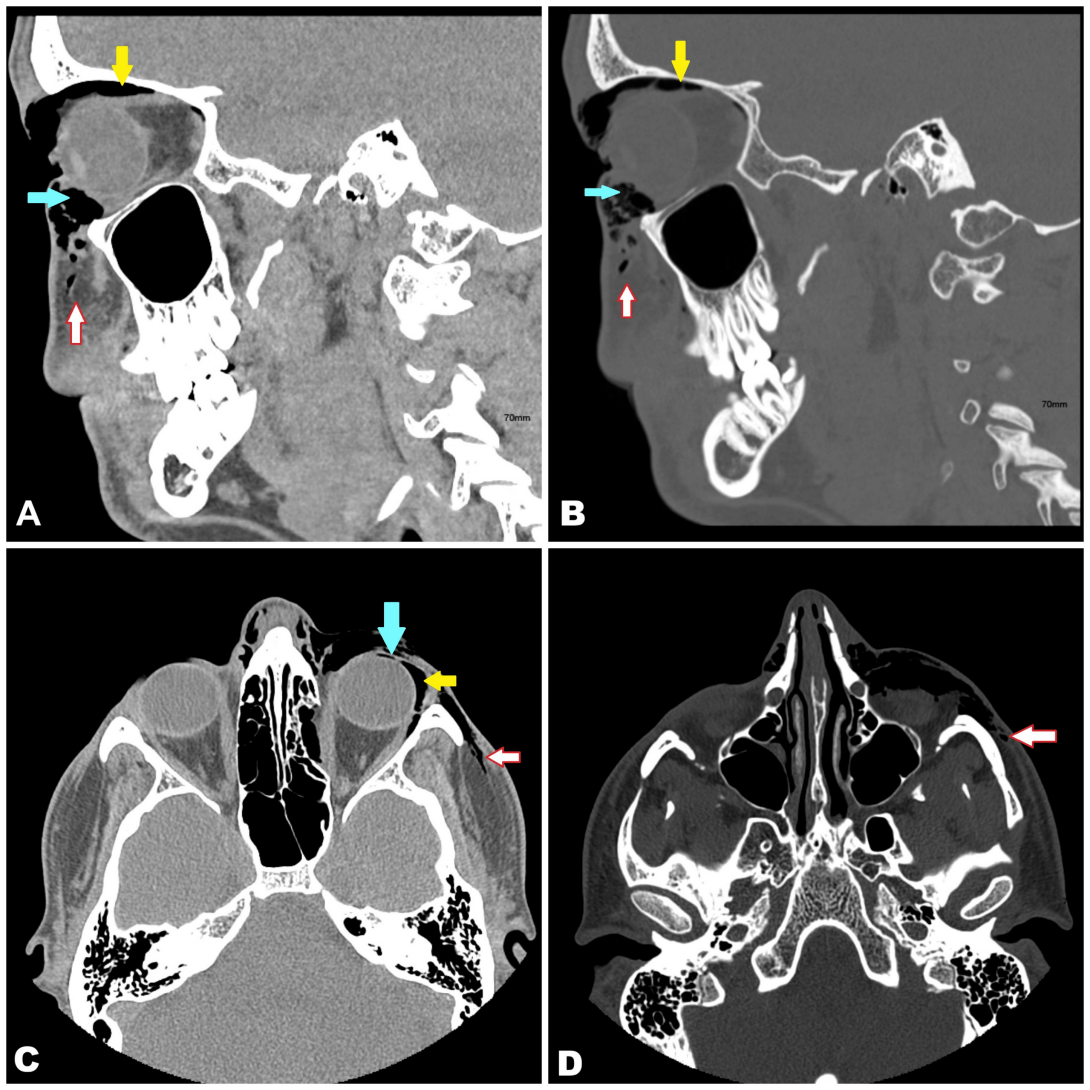
Sagittal (A and B) and axial (C and D) computed tomography (CT) scans of the face demonstrating orbital (yellow arrows), subconjunctival (blue arrows), and subcutaneous emphysema (white arrows) The images highlight the extension of air pockets within the orbital and periorbital tissues.

Computed tomography (CT) of the face demonstrated orbital, subconjunctival, and subcutaneous emphysema associated with a small left medial orbital wall fracture, without evidence of extraocular muscle entrapment, fat herniation, or optic nerve compression (Figures 2, 3). Bilateral nasal bone fractures were also identified.

Following multidisciplinary input from the maxillofacial, ophthalmology, and otorhinolaryngology teams, the decision was made to manage the patient conservatively, as there were no signs of visual complications such as orbital compartment syndrome, ischemic optic neuropathy, extraocular muscle entrapment, proptosis, or a relative afferent pupillary defect. He was prescribed prophylactic oral co-amoxiclav 625 mg three times daily for seven days to prevent secondary orbital cellulitis or septic cavernous sinus thrombosis from sinus fracture involvement, along with lubricating eye drops four times daily. The patient was also advised to avoid nose-blowing. Daily follow-up revealed rapid improvement. By day seven, subconjunctival and subcutaneous emphysema had completely resolved, and topical medications were discontinued. The patient remained asymptomatic with no residual deficits.

## Discussion

Orbital emphysema is a well-documented complication of orbital fractures, most frequently associated with medial orbital wall injuries due to their proximity to the ethmoid sinus [1]. Air enters orbital tissues through the sinus wall defects, producing emphysema that may extend into subcutaneous and subconjunctival spaces. Although orbital emphysema is relatively common, occurring in 20-60% cases of orbital fractures, the prevalence of subconjunctival emphysema is far less certain [1]. In medial orbital wall fractures, up to 75% of patients demonstrate orbital emphysema [1].

Simultaneous orbital, subcutaneous, and subconjunctival emphysema is exceptionally rare. To the best of our knowledge, only two prior cases have been reported by Ababneh et al. in a 16-year-old patient and by Boyer et al. in a 42-year-old patient, both following acute orbital floor fractures [5,6]. In our case, the triad developed in a 21-year-old male following blunt orbital trauma, making it among the very few such presentations described in the literature.

Subconjunctival emphysema is an uncommon manifestation, characterized by the presence of air between Tenon’s capsule and the conjunctiva. It is often obscured by more prominent subcutaneous emphysema, which makes clinical detection challenging [1,2]. Subconjunctival emphysema itself does not raise intraorbital pressure but signals a possible sinus-orbital communication. Although generally self-limiting, subconjunctival emphysema can coexist with more significant orbital emphysema and, when identified in patients with blunt facial trauma, indicates underlying orbital and sinus wall fractures [3].

The majority of cases resolve spontaneously within days to weeks [7]. Nevertheless, serious complications can occur if orbital emphysema progresses to orbital compartment syndrome, where increasing intraorbital pressure compromises optic nerve perfusion [8]. If intraocular pressure approaches the ophthalmic artery perfusion pressure, ischemic damage to the optic nerve may occur within one to two hours [9]. Because orbital emphysema can elevate pressure to vision-threatening levels, the finding of subconjunctival emphysema after blunt facial trauma warrants imaging and close monitoring for optic nerve compromise.

In the present case, intraocular pressure in the affected eye was only mildly elevated compared to that in the contralateral eye, with no evidence of orbital compartment syndrome, extraocular muscle entrapment, proptosis, relative afferent pupillary defect, optic nerve compromise, or any visual disturbance. Conservative management, including prophylactic oral antibiotics, topical ocular medications, and avoidance of nose-blowing, was sufficient, resulting in complete resolution within one week.

Several non-traumatic and iatrogenic causes of subconjunctival emphysema have also been reported, highlighting the variety of clinical contexts in which this entity can arise (Table 1). Sarbay et al. described subconjunctival emphysema following lung volume reduction surgery in a patient with COPD, which was successfully managed with chest drain suction, oxygen, and needle decompression [10]. Garcia et al. reported subconjunctival and subcutaneous emphysema after rupture of a pulmonary bulla, while Philip et al. described bilateral subconjunctival emphysema associated with pneumomediastinum and subcutaneous emphysema after blunt chest trauma [11,12]. Lee et al. documented pneumocephalus with periorbital and subconjunctival emphysema following comminuted fractures of the orbital floor and maxillary wall [13]. These reports reinforce that while traumatic orbital fractures are the most common cause, subconjunctival emphysema can also occur in systemic and thoracic contexts.

**Table 1:** Reported cases of subconjunctival emphysema with orbital and/or subcutaneous involvement.

Authors	Age	Etiology	Underlying Cause	Clinical Presentation	Management
Ababneh et al. [5]	16 years	Trauma	Orbital floor fracture	Orbital, subcutaneous, and subconjunctival emphysema	Conservative (observation, antibiotics, avoidance of Valsalva)
Boyer et al. [6]	42 years	Trauma	Orbital floor fracture	Orbital, subcutaneous, and subconjunctival emphysema	Conservative
Lee et al. [13]	35 years	Trauma	Orbital floor and maxillary wall fractures	Pneumocephalus, periorbital, and subconjunctival emphysema	Conservative
Sarbay et al. [10]	64 years	Non-traumatic	Post–lung volume reduction surgery in COPD	Subconjunctival emphysema with subcutaneous emphysema	Chest drain suction, oxygen, needle decompression
Garcia et al. [11]	58 years	Non-traumatic	Ruptured pulmonary bulla	Subconjunctival and subcutaneous emphysema	Conservative
Philip et al. [12]	27 years	Trauma	Blunt chest trauma with pneumomediastinum	Bilateral subconjunctival emphysema + subcutaneous emphysema	Conservative
Present case	21 years	Trauma	Medial orbital wall and nasal bone fractures	Orbital + subcutaneous + subconjunctival emphysema	Conservative (oral antibiotics, topical ocular therapy, avoidance of nose-blowing)

Treatment is usually conservative, as air is gradually reabsorbed. Supportive measures include topical lubricants and, in severe cases of lagophthalmos, eyelid taping or conjunctival decompression [7,8]. In rare instances of vision-threatening orbital compartment syndrome, urgent surgical decompression may be required [8,9]. Options include lateral canthotomy and cantholysis, needle aspiration of orbital air, or surgical bony decompression [14,15].

Our case adds to the limited body of evidence reporting the simultaneous presence of orbital, subcutaneous, and subconjunctival emphysema following orbital wall fracture. It emphasizes the importance of early recognition, thorough ophthalmologic and radiologic evaluation, vigilant monitoring of intraocular pressure, and the effectiveness of conservative management in the absence of optic nerve compromise.

## Conclusions

Simultaneous orbital, subcutaneous, and subconjunctival emphysema following blunt facial trauma is an uncommon clinical entity, with only a few cases reported in the literature. Recognition of this triad is important, as it is often associated with orbital wall fractures and may serve as an early clinical marker of more extensive orbital injury. Although most cases are benign and resolve spontaneously, there remains a risk of vision-threatening complications, particularly orbital compartment syndrome and ischemic optic neuropathy resulting from elevated intraorbital pressure.

This case emphasizes the need for a thorough ophthalmic and radiologic assessment in all patients presenting with subconjunctival and periocular emphysema. Continuous monitoring of intraocular pressure and close follow-up are essential to prevent missed progression to optic nerve compromise. Conservative therapy remains effective in uncomplicated cases. Ultimately, this report contributes to the limited literature on simultaneous orbital, subcutaneous, and subconjunctival emphysema and reinforces the importance of early recognition, multidisciplinary evaluation, and vigilant monitoring to ensure optimal visual outcomes.
